# The association between the vitamin B1 intake and risk of non-melanoma skin cancer in U.S. adults: a cross-sectional study

**DOI:** 10.3389/fnut.2025.1571991

**Published:** 2025-04-30

**Authors:** Shiyi Huang, Lungang Shi, Yang Ma, Yuebin Zhu, Yingshi Liang, Haofeng Huang

**Affiliations:** Department of Plastic Surgery, Affiliated Meizhou Hospital of Shantou University Medical College, Meizhou, China

**Keywords:** vitamin B1, non-melanoma skin cancer, NHANES, dietary, cross-sectional study

## Abstract

**Background:**

The incidence of non-melanoma skin cancer (NMSC) has been steadily increasing in recent years, primarily due to global climate change and heightened exposure to ultraviolet (UV) radiation. This trend is particularly pronounced among Caucasians, whose lack of melanin in skin cells renders them more susceptible to UV-induced damage. Although NMSC is associated with low mortality, its high incidence imposes a significant economic burden on society. Consequently, identifying novel risk factors and predictors has become increasingly important. Growing attention has been directed toward the relationship between diet and diseases, including cancer. While research on dietary factors and NMSC has gained momentum, studies specifically examining the link between vitamin B1 intake and NMSC remain limited. Therefore, this study aims to explore the potential association between vitamin B1 consumption and the risk of developing NMSC.

**Methods:**

The authors conducted a cross-sectional study utilizing data from six cycles of the National Health and Nutrition Examination Survey (NHANES) database spanning 2005 to 2016. Out of 60,936 participants initially identified, 30,982 individuals who did not meet the study criteria were excluded, resulting in a final sample of 29,954 participants. In this study, the exposure variable was vitamin B1 intake. This was calculated by counting the amount of vitamin B1 contained in food consumed over a 24-h period. Outcome variable was presence of non-melanoma skin cancer. For the analysis of bivariate variables, multiple linear regression models were primarily employed. Additional analyses included curve fitting and subgroup analysis to further explore the relationships and trends.

**Results:**

The mean age of participants included in the analysis was 49.66 years, with an approximately equal male-to-female ratio. Among these, 499 individuals were diagnosed with NMSC, with a mean age of 68.27 years and a prevalence rate of 1.67%. The probability of having NMSC was positively correlated with vitamin B1 consumption, according to logistic regression analysis. Specifically, an 11% increased incidence of NMSC was linked to every unit increase in vitamin B1 consumption. The risk rose by up to 1.65 times in those with high vitamin B1 consumption, making this link more noticeable. A linearly positive relationship between vitamin B1 consumption and NMSC risk was found using smoothed curve fitting. Subgroup analyses confirmed that this positive association remained consistent across different population subgroups.

**Conclusion:**

This study identified a positive association between vitamin B1 intake and NMSC. However, due to the inherent limitations of cross-sectional studies, further prospective studies are needed to confirm the causal relationship.

## 1 Introduction

Skin cancers encompass various types, including melanoma, basal cell carcinoma (BCC), and squamous cell carcinoma (SCC), among others. The latter two are collectively referred to as NMSC. The most common of them is BCC, which is followed by SCC. Every year, about 18,000 cases of SCC and 36,000 cases of BCC are detected in the US (1). In the United Kingdom, data from 2015–2017 revealed an annual average of approximately 152,000 new NMSC cases, representing a 1.7-fold increase compared to the 1990s (2). Globally, non-melanoma skin cancers account for over 5,400 deaths each month (3). The incidence of skin cancer is projected to rise further, driven by an aging global population and heightened ultraviolet radiation exposure resulting from climate change (4).

Actually, UV radiation disrupts the redox balance of the skin, triggering inflammation and immunosuppression, thereby promoting the development of NMSC. And after the development of NMSC, biomolecules released from the tumor microenvironment tend to exacerbate systemic damage, such as abnormal oxidative stress markers, inflammation and immune dysregulation, and disturbances in vitamin D and calcium metabolism, which may even lead to an increased risk of secondary malignancies (5).

Thankfully, BCC rarely metastasizes due to its tendency for localized growth (6). Although SCC is more aggressive than BCC, most cases are curable with early intervention (7). Consequently, NMSC generally has a low mortality rate, highlighting the importance of early diagnosis and prompt treatment. Despite its lower lethality, the rising incidence of NMSC significantly contributes to the societal healthcare burden. Between 2016 and 2018, the average total annual healthcare expenditure for skin cancer treatment in the United States reached approximately $8.9 billion, with NMSC accounting for about 73% of these costs, i.e., approximately $6.5 billion (8).

In addition to direct medical expenses, NMSC imposes considerable indirect economic burdens, primarily due to work absenteeism and lost productivity (9). Elderly and immunosuppressed patients are particularly prone to prolonged recovery periods, exacerbating work absences. NMSC commonly develops in sun-exposed areas such as the face, head, neck, and ears, where surgical removal often results in scarring. Scars in these visible regions can compromise both appearance and function, leading to psychological distress and additional mental health-related treatment costs (10).

To alleviate the societal burden, preventive measures targeting NMSC are urgently needed. While known risk factors, such as UV radiation, advanced age, and family history, are well-established (11), it remains critical to identify novel risk factors and predictive biomarkers. The discovery of new predictors could enhance the accuracy of early screening, enable personalized treatment approaches, improve prognostic evaluations, and ultimately mitigate the social and economic impacts of NMSC.

The modern world is increasingly health-conscious, driven by the widespread use of the Internet and social media, which have made the concept of healthy eating more accessible and pervasive. As an old Chinese proverb states, “bread is the staff of life,” focusing on the three essential daily meals may provide a promising avenue for identifying novel predictors of health outcomes. In recent years, growing awareness of the pivotal role of diet in disease prevention and quality-of-life improvement has spurred intensified research into the relationship between diet and disease, particularly cancer.

The link between diet and cancer has garnered significant attention (12, 13). Studies suggest that specific foods and dietary patterns are associated with the risk of certain cancers (14–16). This growing body of evidence has not only raised public awareness about healthy eating but has also emphasized the potential of dietary interventions in clinical prevention. In a comprehensive review by Carlos et al., it was systematically discussed how dietary interventions can influence various tumorigenic processes, including nutrient metabolism, growth signaling, antitumor immunity, diet-microbiota interactions, and more (17).

Research into the relationship between diet and NMSC is also advancing. Postmenopausal women, a population at high risk for NMSC, have been the focus of studies linking dietary factors to cancer risk. For instance, one study discovered that postmenopausal women’s dietary consumption of vitamin A and β-cryptoxanthin was linked to their chance of developing NMSC (18). Additionally, a review analyzing 43 studies concluded that dietary folate, citrus, and alcohol intake were positively associated with the risk of BCC, while caffeine intake was inversely associated with BCC risk (19). The anticancer properties of grape seed proanthocyanidins (GSP) have also been demonstrated in experimental NMSC models (20).

Despite these advancements, many aspects of the diet-NMSC relationship remain unexplored. Further investigation into dietary factors and their potential as predictors of NMSC risk could lead to enhanced prevention strategies and a deeper understanding of the interplay between nutrition and skin cancer.

Thiamine, another name for vitamin B1, is a water-soluble vitamin that the body cannot produce on its own and must be acquired from diet. Foods including whole grains, legumes, nuts, and pork are plentiful in it (21). According to the latest data from the USDA’s Food and Nutrient Database for Dietary Studies (FNDDS), every 100 g of pork contains about 0.342 g of vitamin B1 and every 100 g of oatmeal contains 1.431 g of vitamin B1 (22). Vitamin B1 is essential for human health, particularly in energy metabolism, nervous system function, and the normal functioning of cells. Beyond its role in maintaining physiological homeostasis, emerging evidence suggests a potential link between vitamin B1 and tumor biology.

Vitamin B1 is an essential coenzyme in the metabolism of carbohydrates and is involved in vital metabolic processes including the tricarboxylic acid cycle and glycolysis (23). Studies indicate that tumor cells may rely on elevated levels of vitamin B1 to sustain their high metabolic activity (24). For instance, triple-negative breast cancer has been shown to exhibit a strong dependence on vitamin B1 metabolism (25). Insufficient vitamin B1 intake may hinder tumor cell metabolism, thereby inhibiting tumor growth. On the other hand, a greater risk of bladder cancer has been linked to larger intakes of vitamins B1 and B12 (26). Similarly, a Mendelian randomization study reported that higher daily intakes of beta-carotene and vitamin B1 were linked to an elevated likelihood of prostate cancer in men over 40 years of age (27).

However, conflicting evidence exists regarding the relationship between vitamin B1 and cancer. According to Liu et al., a higher risk of colorectal cancer may be linked to a decreased vitamin B1 consumption (28). The underlying mechanisms may involve the antioxidant properties of vitamin B1, its modulation of immune function, and other protective pathways (29, 30). These findings highlight the complexity of vitamin B1’s role in tumorigenesis and underscore the need for further research to clarify its impact across various cancers.

Interestingly, not much research has been done on the connection between vitamin B1 and NMSC. To address this gap, the authors conducted a series of analyses utilizing extensive data from the NHANES database. In the NHANES database, the staff counted the types and weights of food eaten by the volunteers over a 24-h period and used the USDA’s Food and Nutritional Databases for Dietary Studies (FNDDS) to calculate the amount of vitamin B1 contained in each food item. This effort aims to advance understanding in this relatively unexplored area and offer fresh perspectives on vitamin B1’s function in NMSC.

## 2 Materials and methods

The NHANES is a nationally representative, cross-sectional study conducted by the National Center for Health Statistics (NCHS) under the Centers for Disease Control and Prevention (CDC). Since its inception in the 1960s, NHANES has been conducted biennially to systematically collect comprehensive data on the health and nutritional status, as well as related behaviors, of the U.S. ambulatory population. Data collection is achieved through a combination of structured questionnaires, physical examinations, and laboratory tests. The NHANES uses a sophisticated, stratified, multistage probability sampling method to guarantee national representativeness. All collected data are publicly accessible via the official NHANES website.

### 2.1 Sources of data

**Figure 1:**
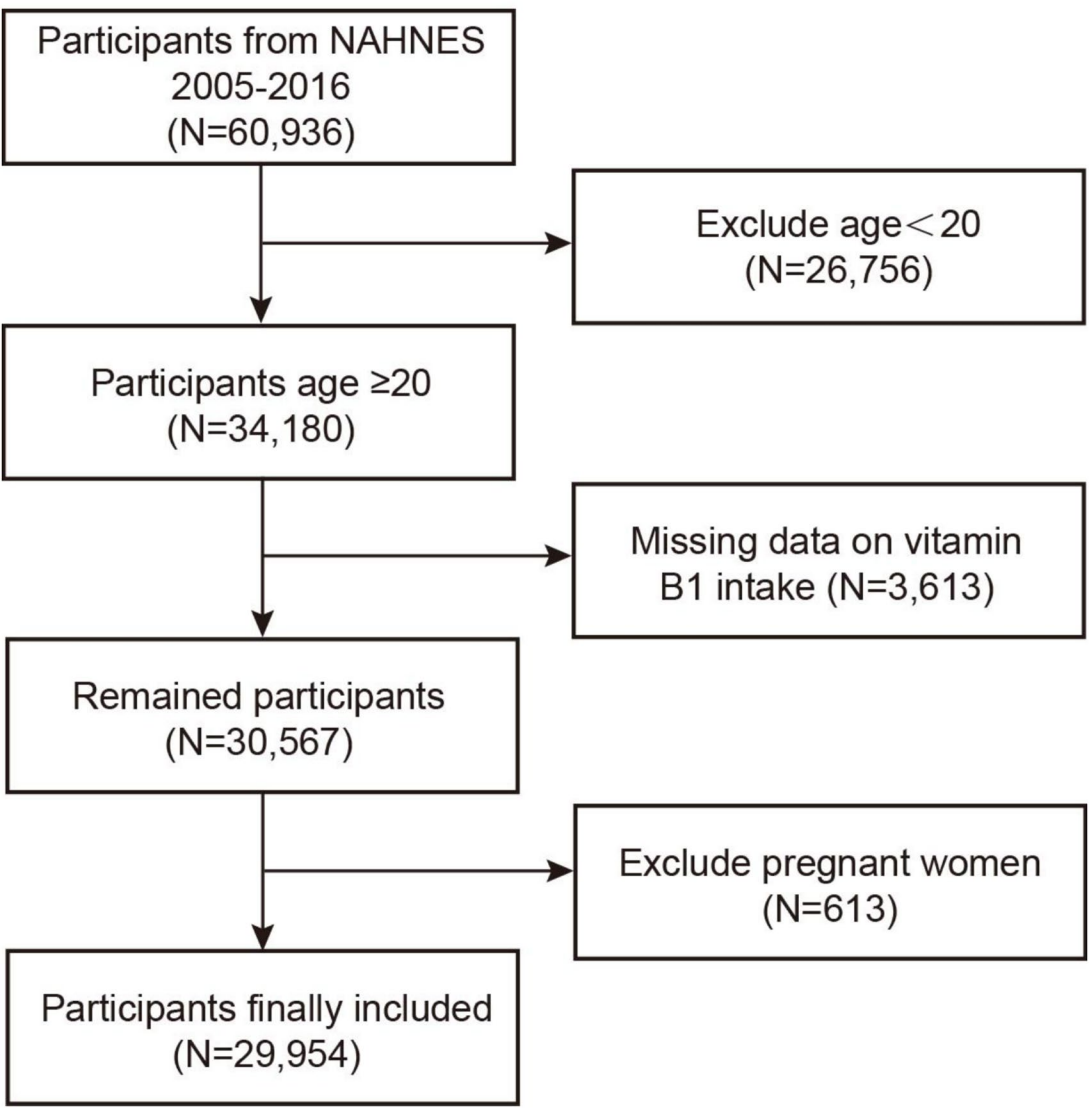
Participant selection flow chart.

This study utilized data from six NHANES cycles conducted between 2005 and 2016, comprising a total of 60,936 participants. Of these, 26,756 individuals under the age of 20 were excluded, as minors have a low probability of developing NMSC, and cases of skin cancer in this population are typically attributed to genetic predispositions or medical exposures (31). Additionally, participants with missing data on vitamin B1 intake and pregnant women were excluded, amounting to 4,226 individuals. Following these omissions, 29,954 individuals made up the final analytical sample (see Figure 1).

### 2.2 Exposure factor

The primary exposure factor in this study was vitamin B1 intake. Research exploring the relationship between vitamin B1 and cancer dates back to the 1940s (32). In the NHANES dietary data section, a 24-h dietary recall is used to record vitamin B1 consumption. During face-to-face interviews, participants are provided with a variety of tools, including bowls, cups, bottles, household spoons, measuring cups and spoons, rulers, and thickness rods, to accurately estimate and report food quantities consumed. After collecting the preliminary data, trained staff process the information using the USDA’s Food and Nutrient Database for Dietary Studies, which calculates nutrient intake and determines the nutritional values of the reported foods.

### 2.3 Outcome factor

Data on NMSC were obtained from the medical condition section of the NHANES questionnaire. Participants were initially asked whether they had ever been diagnosed with cancer by a physician or other healthcare professional. For those who responded affirmatively, additional questions were asked to identify the specific type of cancer. In the dataset, the code “32” represented “Non-melanoma Skin Cancer,” which was designated as the target outcome for this study. To facilitate analysis, the code “32” was recoded as “1,” while all other responses were recoded as “0.”

### 2.4 Covariates

In addition to the two primary variables mentioned above, several covariates were included in this study to adjust for potential confounders in subsequent analyses. Continuous variables such as age, family income-to-poverty ratio (PIR), and BMI were analyzed. Family PIR is commonly used as an indicator of participants’ socioeconomic status. Categorical variables included gender, race, education level, and marital status. Lifestyle factors such as smoking and alcohol use history were also considered. Participants were asked whether they had consumed more than 12 alcoholic drinks per year or smoked 100 cigarettes or more over their lives. Additionally, co-morbidities such as hypertension and diabetes were incorporated, with participants reporting whether a healthcare professional had ever diagnosed them with these conditions.

As for dietary factors, apart from the primary exposure variable, the use of dietary supplements and 24-h energy intake were included. In subsequent analyses, continuous variables were categorized for ease of interpretation. Age was grouped into three categories; family PIR was classified as <1.3, ≥1.3 but <3.5, and ≥3.5; BMI was categorized as <30 kg/m^2^ or ≥30 kg/m^2^; and energy intake was dichotomized into high-energy and low-energy groups.

### 2.5 Statistical analysis

R software (version 4.2) and EmpowerStats (version 2.0), which are both frequently used for statistical computing and graphical representation, were used for all statistical analyses in this work. *p*-values below 0.05 were regarded as statistically significant. The median of the continuous variables or the plural of the categorical variables for instances of these variables that already exist are used to insert missing values. For the descriptive analysis of demographic characteristics, weighted Student’s *t*-tests were employed for continuous variables, with results expressed as mean ± standard error. For categorical variables, weighted chi-square tests were used, with results presented as percentages.

To investigate the independent association between vitamin B1 intake and NMSC, multivariate regression models were utilized. Confounding variable modifications were absent from Model 1. While Model 3 was corrected for age, race, education status, alcoholic status, dietary supplement use and family PIR; Model 2 was adjusted for age, race, and education level. For the variables adjusted in model 3, they were screened by stepwise regression analysis (two-way method) (*p* < 0.05). The results of the multicollinearity analysis showed that the variance inflation factors (VIF) of the independent variables in Model 3 were less than 5, suggesting that the collinearity between the independent variables was not significant. In order to test for trends, sensitivity analyses converted vitamin B1 consumption from a continuous variable into a four-category variable. To better clarify any possible linear interactions, the trend between vitamin B1 consumption and NMSC was further shown using weighted generalized additive model (GAM) regression approaches and smoothed curve fitting. To assess the stability of the connection across different subgroups, stratified by all relevant factors, subgroup analyses were performed using interaction tests.

## 3 Results

### 3.1 Baseline characteristics of participants

This research includes 29,954 individuals, with a weighted mean age of 49.66 years. Participants with NMSC had a mean age of 68.27 years, which was 18.93 years higher than those without NMSC. This finding underscores the elevated risk of non-melanoma skin cancer among older adults. The male-to-female ratio in the study was approximately equal, while the NMSC population had a slightly larger percentage of men. This aligns with the known epidemiological patterns of non-melanoma skin cancer (33), which may be attributed to lifestyle factors such as increased outdoor activities and higher UV exposure among males.

The overall mean vitamin B1 intake among all participants was 1.58 mg, and the prevalence of NMSC was 1.67%. Participants with NMSC tended to be older, male, and non-Hispanic Caucasian, consistent with established epidemiological characteristics (33). Additionally, individuals with NMSC were more likely to have higher educational attainment, consume alcohol and tobacco, have hypertension, use dietary supplements, have higher vitamin B1 intake, be married, possess a higher family income-to-poverty ratio, and have a lower BMI (all *p* < 0.05).

The associations with high education levels and higher family income-to-poverty ratios may reflect greater financial means to engage in outdoor activities (e.g., golf, tennis, skiing, and beach vacations), which often lead to increased sun exposure. Similarly, lower BMI could suggest a more active lifestyle with frequent outdoor exercise, thereby increasing UV exposure, a well-established risk factor for NMSC. These observations help explain the heightened risk of NMSC in these populations.

In contrast, no significant differences in the prevalence of NMSC were observed in subgroups defined by diabetes status or energy intake. All individuals’ baseline characteristics are shown in Table 1, stratified by NMSC status. Notably, the higher vitamin B1 intake observed in the NMSC group compared to the NMSC group prompted further investigation into the relationship between vitamin B1 intake and NMSC.

**Table 1:** Baseline characteristics of participants between 2005 and 2016 (*n* = 29,954).

Characteristics	Non-skin cancer (*N* = 29,455)	Skin cancer (*N* = 499)	*P*-value
Age (years)	49.34 ± 17.69	68.27 ± 12.61	<0.001
Gender, *n* (%)			
Male	14,556 (49.42%)	281 (56.31%)	
Female	14,899 (50.58%)	218 (43.69%)	
Race/ethnicity, *n* (%)			<0.001
Mexican American	4,744 (16.11%)	6 (1.20%)	
Other Hispanic	2,856 (9.70%)	9 (1.80%)	
Non-Hispanic White	12,696 (43.10%)	472 (94.59%)	
Non-Hispanic Black	6,393 (21.70%)	5 (1.00%)	
Other race	2,766 (9.39%)	7 (1.40%)	
Education level, *n* (%)			<0.001
Less than 9th grade	3,234 (10.98%)	25 (5.01%)	
9-11th grade	4,357 (14.79%)	37 (7.41%)	
High school graduate	6,787 (23.04%)	90 (18.04%)	
Some college or AA degree	8,526 (28.95%)	160 (32.06%)	
College graduate or above	6,551 (22.24%)	187 (37.47%)	
Smoked at least 100 cigarettes, *n* (%)			<0.001
Yes	13,315 (45.20%)	276 (55.31%)	
No	16,140 (54.80%)	223 (44.69%)	
Hypertension, *n* (%)			<0.001
Yes	10,542 (35.79%)	273 (54.71%)	
No	18,913 (64.21%)	226 (45.29%)	
Diabetes, *n* (%)			0.085
Yes	3,730 (12.66%)	71 (14.23%)	
No	25,090 (85.18%)	411 (82.36%)	
Borderline	635 (2.16%)	17 (3.41%)	
Dietary supplement use	1.15 ± 2.30	2.29 ± 2.73	<0.001
Energy intake (kcal)	2,101.41 ± 1002.5	1,985.72 ± 747.7	0.179
Vitamin B1 intake (mg)	1.58 ± 0.93	1.68 ± 0.83	0.016
Had at least 12 alcohol drinks/year, *n* (%)			<0.001
Yes	19,739 (71.43%)	378 (78.42%)	
No	7,895 (28.57%)	104 (21.58%)	
Marital status, *n* (%)			<0.001
Married	15,124 (51.35%)	317 (63.53%)	
Widowed	2,359 (8.01%)	82 (16.43%)	
Divorced	3,214 (10.91%)	60 (12.02%)	
Separated	1,000 (3.40%)	9 (1.80%)	
Never married	5,407 (18.36%)	20 (4.01%)	
Living with partner	2,351 (7.98%)	11 (2.20%)	
Family PIR	2.50 ± 1.56	3.27 ± 1.51	<0.001
BMI (kg/m^2^)	29.14 ± 6.87	28.09 ± 5.61	<0.001

Mean ± SD for continuous variables: the *P* value was calculated by the weighted linear regression model. (%) for categorical variables: the *P* value was calculated by the weighted chi-square test.

Family PIR, the ratio of family income to poverty; BMI, body mass index.

### 3.2 Vitamin B1 and NMSC

The level of vitamin B1 intake over a 24-h period was analyzed as a continuous variable in a logistic regression model to evaluate its association with NMSC. The risk of NMSC was positively correlated with vitamin B1 consumption, as seen in Table 2. In model 1, which did not adjust for any confounders, each 1-unit increase in vitamin B1 intake was associated with an 11% higher risk of developing NMSC (OR: 1.11, 95% CI: 1.02–1.20). This positive association persisted after adjusting for partial confounders (model 2, OR: 1.16, 95% CI: 1.06–1.27) and further adjustments for 6 covariates (model 3, OR: 1.14, 95% CI: 1.03–1.26).

**Table 2:** Association of vitamin B1 intake with non-melanoma skin cancer.

	OR (95% CI)		
Exposure	Model 1	Model 2	Model 3
	(*n* = 29,954)	(*n* = 29,954)	(*n* = 25,967)
Vitamin B1	1.11 (1.02, 1.20)	1.16 (1.06, 1.27)	1.14 (1.03, 1.26)
Vit B1 quartile			
Quartile 1	Reference	Reference	Reference
Quartile 2	1.55 (1.18, 2.05)	1.32 (1.00, 1.76)	1.38 (1.02, 1.86)
Quartile 3	1.85 (1.41, 2.42)	1.49 (1.13, 1.97)	1.49 (1.11, 2.00)
Quartile 4	1.65 (1.25, 2.17)	1.65 (1.24, 2.19)	1.58 (1.17, 2.15)
P for trend	0.001	<0.001	0.006

In sensitivity analysis, vitamin B1 intake was converted from a continuous variable to a categorical variable (quartile).

Model 1: No covariates were adjusted.

Model 2: Age, race/ethnicity and education level were adjusted.

Model 3: Age, race/ethnicity, educational level, Family PIR, drinking alcohol status and dietary supplement use were adjusted.

OR, odds ratio; 95% CI, 95% confidence interval; Family PIR, the ratio of family income to poverty.

The consumption of vitamin B1 was divided into quartiles (Q1–Q4) in order to confirm the trend of this connection. Participants in Q4 showed a 1.65-fold higher risk of NMSC than those in Q1 (model 2, OR: 1.65, 95% CI: 1.24–2.19). This trend remained significant after further adjustment (model 3, OR: 1.58, 95% CI: 1.17–2.15).

### 3.3 Smoothed curve fit and subgroup analysis

**Figure 2:**
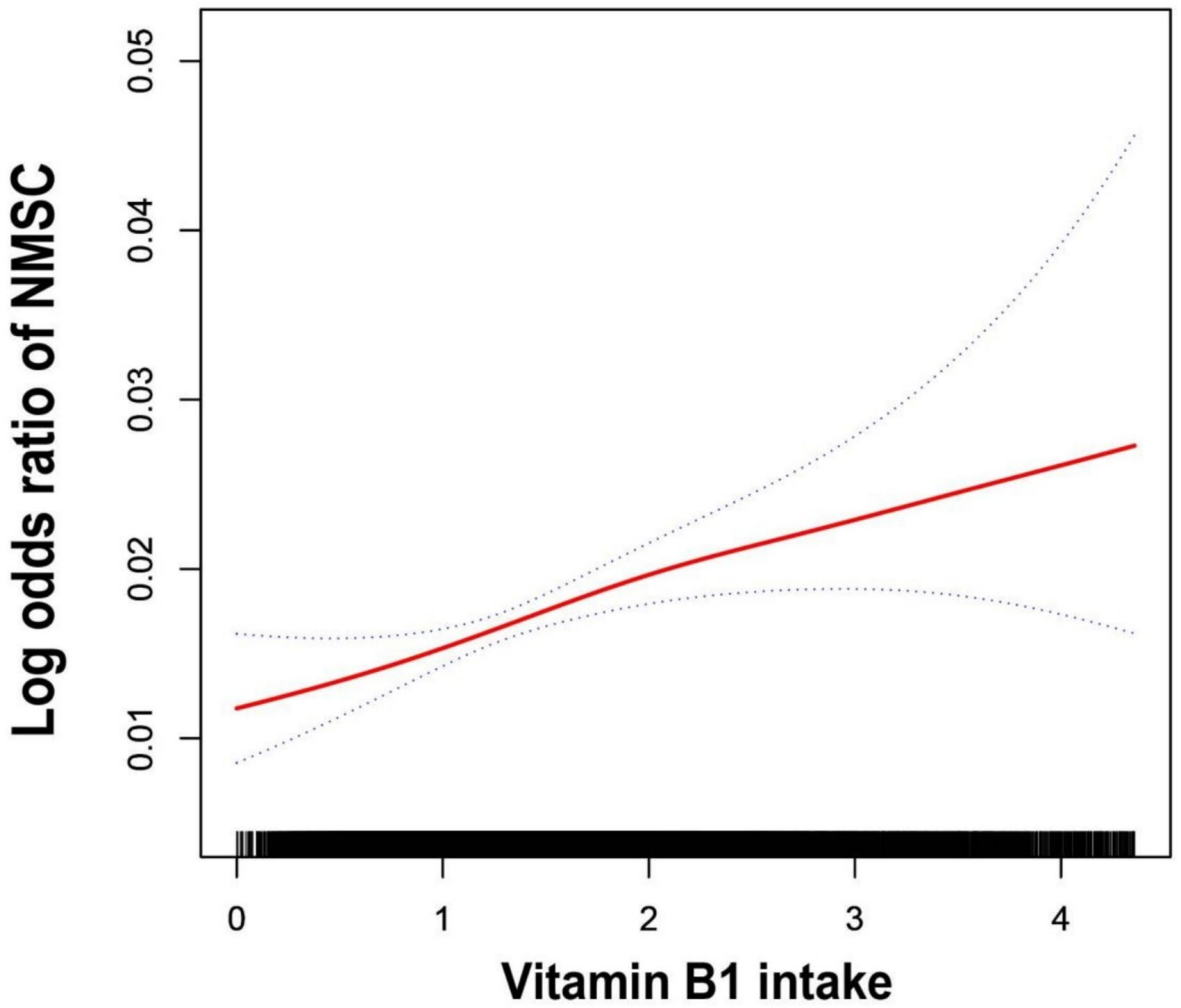
The generalized additive model fits a smooth curve. The fitted curve was indicated by the red line, and 95% confidence intervals were shown by the two blue lines. Model adjusted for age, race/ethnicity, educational level, family PIR, drinking alcohol status and dietary supplement use.

**Figure 3:**
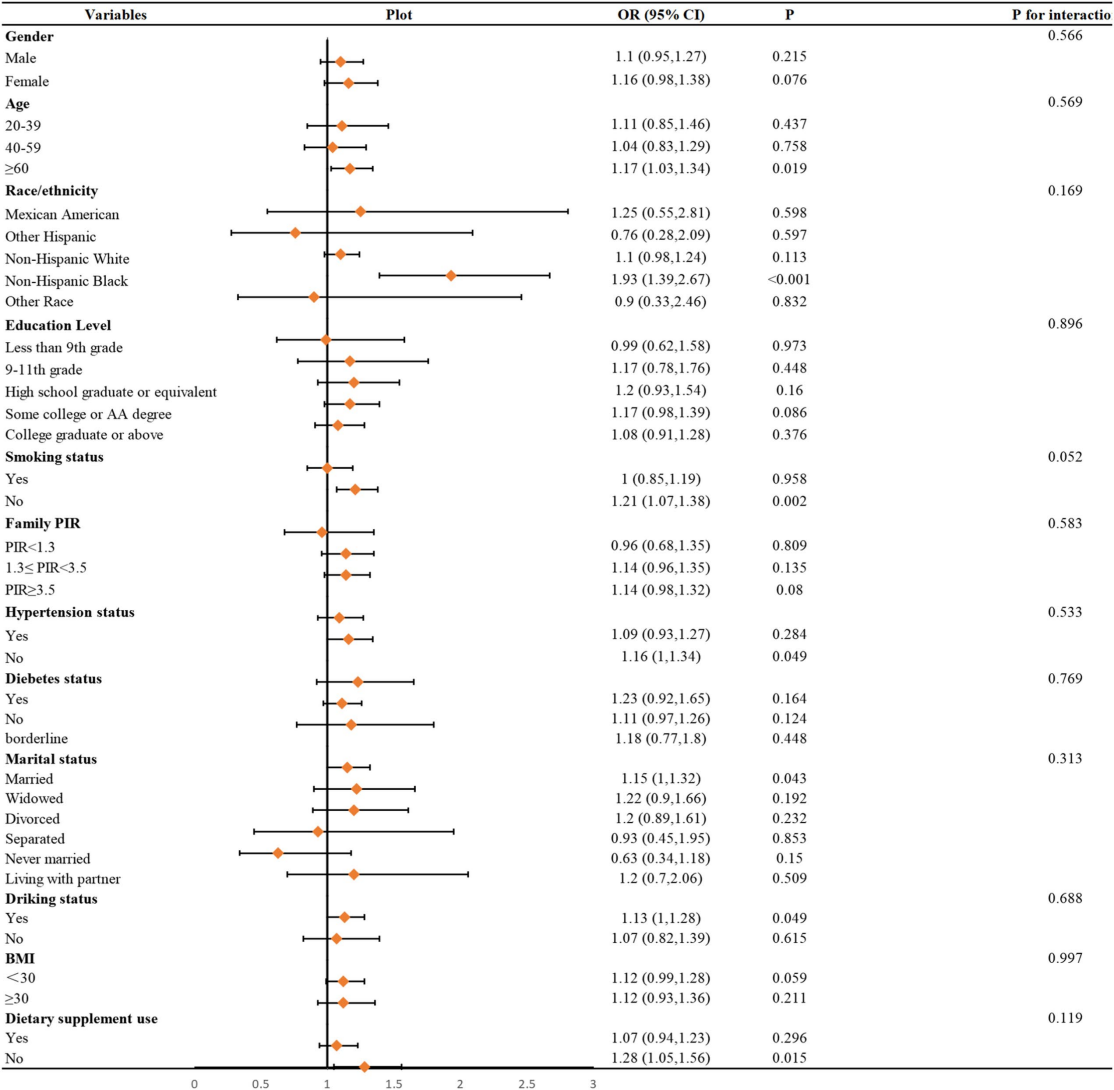
Subgroups analyses. In the subgroup analysis stratified by age, gender, race/ethnicity, educational level, family PIR, hypertension status, BMI, smoking status, drinking alcohol status, diabetes, dietary supplement use, daily energy intake, and the model is not adjusted for age, gender, race/ethnicity, educational level, family PIR, hypertension status, BMI, smoking status, drinking alcohol status, diabetes, dietary supplement use, daily energy intake, respectively.

To further illustrate the relationship, the authors used smoothed curve fitting to visualize the trend (Figure 2). The findings showed a linearly increased relationship between NMSC risk and vitamin B1 consumption. To evaluate the stability of this connection across other factors, subgroup analyses were also conducted. As shown in Figure 3, no significant between-group differences were observed in any subgroup (*p* > 0.05). This suggests that there was a strong and constant positive correlation between vitamin B1 consumption and NMSC across all different population strata.

## 4 Discussion

The authors identified a positive association between vitamin B1 intake and NMSC through a cross-sectional study utilizing a large, publicly available U.S. database. This finding provides further insight into the role of vitamin B1 in cancer development. Additionally, a trend test revealed that the association between vitamin B1 intake and NMSC risk strengthened as vitamin B1 intake increased. The curve fitting visually shows us the trend in vitamin B1 intake and suffering from NMSC. Subgroup analyses showed that this relationship was stable across various subgroups, enhancing the generalizability of the study’s findings.

In fact, there have been many studies on the correlation between vitamins and skin cancer. For example, Seretis et al. concluded, based on 10 systematic reviews, that high Vitamin D serum levels may indirectly increase the risk of NMSC through sun exposure. And Vitamin D supplementation showed a potential protective effect in some studies (34). Therefore, dietary supplementation is recommended as an alternative to sun exposure for obtaining Vitamin D. In addition, nicotinamide, a derivative of vitamin B3, prevents photoaging of the skin and reduces the incidence of skin cancer (35). Vitamin A intake, on the other hand, has been found to be positively associated with the risk of NMSC in postmenopausal women (18).

Similarly, vitamin B1 is also an important vitamin in the body. The relationship between vitamin B1 and NMSC is being investigated for the first time in this study, thereby bridging a significant research gap and reaffirming the critical role of dietary factors in NMSC risk. From a clinical perspective, these findings suggest that physicians should consider limiting excessive vitamin B1 intake during nutritional counseling, particularly in dietary recommendations for individuals at high risk of NMSC. Indeed, Table 1 shows that the average age of the control group was 49 years and the average age of the NMSC group was 69 years. This difference in age suggests that UV radiation-induced mutations are likely to accumulate over a period of time eventually leading to senile NMSC. Therefore, attention should be given to patients with a family history of cancer, occupational UV exposure, and the elderly.

The cross-sectional form of the study, however, restricts its capacity to prove causation. Future research, including basic studies on the effects of varying doses of thiamine pyrophosphate (TPP), the active form of vitamin B1, on the growth of NMSC cells, as well as prospective studies, is needed to confirm the causal relationship between vitamin B1 intake and NMSC.

The potential mechanisms underlying thiamine’s role in carcinogenesis remain poorly understood, necessitating further investigation by researchers. Currently, it is hypothesized that its carcinogenic effects may primarily arise from its support of the energy demands required for tumor cell proliferation. Thiamine, in its active form as TPP, serves as a coenzyme for several critical enzymes involved in energy metabolism.

One such enzyme is the pyruvate dehydrogenase complex, a multi-enzyme system located in the mitochondrial matrix and a rate-limiting enzyme in cellular aerobic oxidation. This complex catalyzes the irreversible oxidative decarboxylation of pyruvate to acetyl coenzyme A, serving as a vital link between glycolysis, the tricarboxylic acid cycle, and oxidative phosphorylation (29). TPP acts as the essential coenzyme for the proper functioning of the pyruvate dehydrogenase complex. In thiamine deficiency, the lack of TPP impairs the activity of this complex, leading to inefficient conversion of pyruvate to acetyl-CoA, disrupting energy metabolism and potentially resulting in metabolic abnormalities such as lactic acidosis.

TPP is also a coenzyme for the α-ketoglutarate dehydrogenase complex, another key enzyme in the TCA cycle that catalyzes the conversion of α-ketoglutarate to succinyl coenzyme A. These roles underscore the indispensable function of vitamin B1 in maintaining cellular energy metabolism and highlight its potential involvement in cancer progression.

Furthermore, transketolase is a key enzyme in the pentose phosphate pathway. It helps to produce ribulose-5-phosphate and erythrose-4-phosphate during the non-oxidizing phase of the pentose phosphate pathway. On another level, a large number of nucleotides are required for cell replication. And ribulose-5-phosphate is exactly the necessary precursor for synthesizing ribonucleic acid and deoxyribonucleic acid. In rapidly dividing cells such as cancer cells and stem cells, the pentose phosphate pathway activity is enhanced and transketolase expression is upregulated to meet the nucleotide demand. TPP is precisely the cofactor of transketolase. In a study by Comín‐Anduix et al. it was proposed that thiamine supplementation could have the effect of increasing tumor growth in thiamine-deficient advanced tumors (36). This effect was produced through the activation of transketolase by thiamine.

Tumor cells are characterized by an exceptionally high metabolic demand, a phenomenon known as metabolic reprogramming, which is a hallmark of cancer. To support rapid division and proliferation, the metabolic rate of tumor cells is typically 5–10 times higher than that of normal cells, depending on the tumor type and growth stage. Notably, poorly differentiated cancer cells generally exhibit a higher metabolic rate compared to highly differentiated ones. This suggests that adequate thiamine intake may create favorable conditions for the heightened metabolic activity of tumor cells.

In addition to their ability to utilize both aerobic and anaerobic metabolic pathways, tumor cells often exhibit a distinct metabolic phenotype known as the Warburg effect. Even when there is enough oxygen present, tumor cells preferentially use anaerobic glycolysis to produce energy. This phenomenon is a defining characteristic of tumor cell metabolism. The Warburg effect reflects the metabolic adaptation of tumor cells to meet their energy and biosynthetic needs during rapid growth and proliferation.

According to studies, breast cancer cells have substantially greater thiamine levels than do healthy breast epithelial cells (37). Using a chronic hypoxia breast cancer cell line model, Sweet et al. demonstrated that the expression of thiamine transporter proteins increases more than 30-fold in breast cancer cells under hypoxic conditions. The study further highlighted that hypoxic environments often lead to lactate accumulation, which lowers the pH of the tumor microenvironment. This acidic microenvironment promotes tumor invasion and metastasis, suppresses the immune response, and is associated with poor prognosis (38).

Additionally, patients with advanced cancer are often more susceptible to hemorrhagic thiamine deficiency, leading to the frequent use of thiamine as a nutritional supplement in clinical settings. In an experimental study, the team led by Begoña administered stepwise thiamine supplementation to tumor-inoculated mice. When the supplementation dose reached 25 times the recommended intake, tumor growth was stimulated by 1.64-fold. Interestingly, when the dose increased to approximately 2,500 times the recommended intake, the opposite effect was observed, with significant inhibition of tumor cell growth (36).

Thiamine has also been suggested to inhibit tumor growth and exhibit anticarcinogenic properties. A case–control study conducted in 1998 investigated the correlation between gastric cancer risk and nutrient intake. By statistically analyzing data from a middle-aged and elderly population, the study found that nutrients such as vitamin B1, B3, and vitamin C were associated with a decreased risk of gastric cancer (39). Furthermore, reduced thiamine levels in leukocytes were observed in patients with acute-onset leukemia (40).

The potential anticancer properties of vitamin B1 may be attributed to its antioxidant effects. Reactive oxygen species (ROS) and reactive nitrogen species (RNS) are produced in excess of the body’s antioxidant capacity during oxidative stress, which damages DNA in cells. If these mutations accumulate, they may contribute to cancer development. Additionally, cancer cells often thrive in environments of heightened oxidative stress, which not only affects immune cells, blood vessels, and the tumor microenvironment but also promotes cancer cell proliferation, invasion, and metastasis by activating transcription factors such as NF-κB and AP-1. By increasing the production and activity of antioxidant molecules like glutathione (GSH), thiamine, a vital antioxidant factor, can lower intracellular ROS levels (41). This mechanism helps protect normal cells from oxidative damage and may slow cancer cell proliferation and metastasis.

In conclusion, the effects of vitamin B1 on cancer are multifaceted and complex, necessitating further research to elucidate the underlying mechanisms. The authors’ study contributes valuable insights to this growing field of investigation.

The NHANES database employs a nationwide randomized sampling design, ensuring that the samples are representative of the state of the American people’s diet and health. Findings based on NHANES can often be generalized to broader populations. Since its inception in the 1960s, NHANES has accumulated a wealth of historical data, and its sophisticated data collection methods provide a reliable foundation for analyzing trends and changes in health over time.

In the current study, the inclusion of numerous covariates allowed for the adjustment of multiple confounding factors, enhancing the stability and reliability of the findings. Adjusting for confounders through multiple models strengthens the robustness of the results. Additionally, subgroup analyses accounted for population diversity, enabling the evaluation of different subpopulations and increasing the generalizability of the conclusions.

However, due to its cross-sectional design, this study was unable to prove a causal association between vitamin B1 use and NMSC. Future investigations should validate these findings using multicenter databases and confirm causality through prospective studies. And individuals diagnosed with NMSC may have changed their diets due to health problems, resulting in associations of exposure variables with outcome variables that do not reflect the initial risk factors, which in turn leads to reverse causal bias. Moreover, NHANES depends on participant self-reported data, which might introduce recall or reporting bias and result in inaccurate results. Although NHANES includes a broad range of variables to account for potential confounders, the inherent limitations of cross-sectional study designs mean that it may not be possible to completely eliminate residual confounding factors.

## 5 Conclusion

Overall, the authors utilized representative data from the NHANES database and logistic regression analysis to examine the correlation between vitamin B1 intake and the risk of developing NMSC. The results showed that among the study population, a higher vitamin B1 intake was linked to a higher incidence of NMSC. This study provides valuable insights into the potential mechanisms linking diet and cancer and offers a novel perspective for NMSC prevention strategies. However, as a cross-sectional study, it cannot establish causality between vitamin B1 intake and NMSC risk. To elucidate the causative association and further explore the role of vitamin B1 in NMSC, prospective research in the future are crucial.

## Data Availability

Publicly available datasets were analyzed in this study. This data can be found here: NHANES (https://wwwn.cdc.gov/nchs/nhanes/Default.aspx).
